# Genetic screen for suppressors of increased silencing in *rpd3* mutants in *Saccharomyces cerevisiae* identifies a potential role for H3K4 methylation

**DOI:** 10.1093/g3journal/jkab309

**Published:** 2021-09-17

**Authors:** Richard A Kleinschmidt, Laurie M Lyon, Samantha L Smith, Jonah Rittenberry, K Maeve Lawless, Anabelle A Acosta, David Donze

**Affiliations:** 1 Department of Biology, Delgado Community College, New Orleans. LA 70119, USA; 2 Department of Biological Sciences, Louisiana State University, Baton Rouge, LA 70803, USA

**Keywords:** silencing, *RPD3*, *BRE1*, *BRE2*, *SET1*, COMPASS, histone modifications, chromatin, *Saccharomyces cerevisiae*

## Abstract

Several studies have identified the paradoxical phenotype of increased heterochromatic gene silencing at specific loci that results from deletion or mutation of the histone deacetylase (HDAC) gene *RPD3*. To further understand this phenomenon, we conducted a genetic screen for suppressors of this extended silencing phenotype at the *HMR* locus in *Saccharomyces cerevisiae*. Most of the mutations that suppressed extended *HMR* silencing in *rpd3* mutants without completely abolishing silencing were identified in the histone H3 lysine 4 methylation (H3K4me) pathway, specifically in *SET1*, *BRE1*, and *BRE2*. These second-site mutations retained normal *HMR* silencing, therefore, appear to be specific for the *rpd3*Δ extended silencing phenotype. As an initial assessment of the role of H3K4 methylation in extended silencing, we rule out some of the known mechanisms of Set1p/H3K4me mediated gene repression by *HST1*, *HOS2*, and *HST3* encoded HDACs. Interestingly, we demonstrate that the RNA Polymerase III complex remains bound and active at the *HMR*-tDNA in *rpd3* mutants despite silencing extending beyond the normal barrier. We discuss these results as they relate to the interplay among different chromatin-modifying enzyme functions and the importance of further study of this enigmatic phenomenon.

## Introduction

While first discovered in the 1960s (Allfrey *et al.* 1964), histone post-translational modifications became a major focus of chromatin research with the identification of the first histone modification writers and erasers, the tetrahymena *GCN5* ortholog acetyltransferase (Brownell *et al.* 1996) and the *RPD3* ortholog histone deacetylases (HDACs; Taunton *et al.* 1996). Since then, an explosion of discoveries has been made identifying epigenetic writers, erasers, and readers, with demonstration of their roles in numerous chromatin processes including gene transcription and silencing, and DNA replication, repair, and recombination (Allis and Jenuwein 2016).

While early results suggested binary correlation of histone acetylation with active gene expression and deacetylation with repression (Hebbes *et al.* 1988; Johnson *et al.* 1990; Park and Szostak 1990), several studies in *Saccharomyces* *cerevisiae* have shown that loss of function of the HDAC *RPD3* paradoxically leads to suppression of defective silencing or an increase in heterochromatic silencing at all three *SIR* (silent information regulator) dependent loci in yeast (Sussel *et al.* 1995; Rundlett *et al.* 1996; Vannier *et al.* 1996; Smith *et al.* 1999; Sun and Hampsey 1999). Increased silencing in *rpd3* mutants has also been observed in metazoans at telomeres in *Drosophila* (De Rubertis *et al.* 1996). Previous work from our lab showed that mutation of *S. cerevisiae RPD3* led to the spread of silencing beyond the normal tDNA heterochromatin barrier element at the *HMR* locus resulting in silencing of downstream reporter genes (Jambunathan *et al.* 2005), and more recent studies have shown a possibly related restoration of defective yeast *SIR*-mediated silencing by second-site point mutations in *RPD3* (Thurtle-Schmidt *et al.* 2016). Both this study and our unpublished results suggest that this increased silencing is due to loss of function of the RPD3L versus the RPD3S complex. While some explanations put forth for this increased heterochromatic silencing include boundary/barrier activity of Rpd3p (Ehrentraut *et al.* 2010) or redistribution of Sir proteins in *rpd3* mutants (Zhou *et al.* 2009), it is not entirely clear how loss of HDAC activity results in an increase of heterochromatic silencing at specific loci.

To address this question in more detail and identify additional potential effectors of this *rpd3* phenotype, we sought to isolate suppressor mutants that reverse the increased spreading of silencing at *HMR* in *rpd3* mutant strains without completely abolishing silencing as would occur with second-site mutations in the *SIR* genes. *MAT***α** strains containing the *rpd3*Δ::KanMX allele and an *HMR*-*ADE2* silencing reporter gene were subjected to transposon mutagenesis, and isolates that reversed silencing of the ectopic *ADE2* gene but retained a mating phenotype indicative of normal *HMRa* silencing were partially characterized. Of the mutants identified, most were in the histone H3 lysine 4 (H3K4) methylation pathway, with multiple independent hits in *SET1*, *BRE1*, and *BRE2*. We also show results that known mediators of H3K4 repression are not involved and that the RNA Polymerase III complex remains actively bound at the *HMR*-tDNA in *rpd3*Δ strains.

## Materials and methods

Selected transposon mutant strains isolated in the screen that were further characterized in this study are listed in Table 1. All strains described in the figures and their genotypes are listed in Table 2. Oligonucleotides used with brief descriptions are listed in Table 3.

**Table 1:** List of primary transposon insertion mutant strains characterized in this paper

Isolate	Gene	Location	Insertion site	Strain number
L8	*BRE2*	Chr XII	176,534	DDY5690
L11	*NGG1*	Chr IV	814,931	DDY5624
L13	*SET1*	Chr VIII	348,435	DDY5625
MB	*BRE2*	Chr XII	176,241	DDY5626
A4	*BRE1*	Chr IV	324,598	DDY5642
A5	*SET1*	Chr VIII	347,931	DDY5691
A8	*BRE2*	Chr XII	175,842	DDY5692
A35	*SET1*	Chr VIII	348,615	DDY5643
L31	*SIR4*	Chr IV	920,020	DDY5640
M4	*SIR4*	Chr IV	920,651	DDY5641

**Table 2:** *Saccharomyces cerevisiae* strains used and generated in this study

Name	Genotype	Source
DDY20	*MATa his4*	J. Rine
DDY277	*MAT* **α** *his3 leu2 lys2*Δ *trp1 ura3 HMR*ΔI + tDNA Barrier at *a2*	Donze Lab
DDY282	*MAT* **α** *his3 leu2 lys2*Δ *trp1 ura3 HMR*ΔI	Donze Lab
DDY814	*MAT*a *ade2 his3 leu2 trp1 ura3 HMR*-*ADE2*	Donze Lab
DDY465	*MAT*a *his3 leu2 lys2*Δ *trp1 ura3 HMR tRNA*Δ	Donze Lab
DDY466	*MAT* **α** *his3 leu2 lys2*Δ *trp1 ura3 HMR tRNA*Δ	Donze Lab
DDY1344	*MAT* **α** *his3 leu2 lys2*Δ *trp1 ura3 HMR*ΔI-tDNA Barrier-a1 Tn::*LEU2*:*rpd3*	Donze Lab
DDY2973	*MAT* **α** *ade2 his3 leu2 trp1 ura3 HMR*-*ADE2 rpd3*Δ::KanMX	Donze Lab
DDY3133	*MAT* **α** *ade2 his3 leu2 lys2Δ trp1 ura3* VII-L-*URA3*-*TEL ppr1*Δ::*TRP1 HMR*-*ADE2 rpd3*Δ::KanMX	Donze Lab
DDY3396	*MAT*a *his3 leu2 trp1 ura3 sir2*::*TRP1 rpd3*Δ::*KanMX HMR tRNA +19*	Donze Lab
DDY3398	*MAT*a *his3 leu2 lys2*Δ *trp1 ura3 sir2*::*TRP1 HMR tRNA +19*	Donze Lab
DDY3400	*MAT*a *his3 leu2 trp1 ura3 rpd3*Δ::*KanMX HMR tRNA +19*	Donze Lab
DDY3401	*MAT*a *his3 leu2 lys2*Δ *trp1 ura3 HMR tRNA +19*	Donze Lab
DDY3402	*MAT* **α** *his3 leu2 lys2*Δ *trp1 ura3 HMR tRNA +19*	Donze Lab
DDY3403	*MAT* **α** *his3 leu2 trp1 ura3 rpd3*Δ::*KanMX HMR tRNA +19*	Donze Lab
DDY3404	*MAT* **α** *his3 leu2 trp1 ura3 rpd3*Δ::*KanMX HMR tRNA +19*	Donze Lab
DDY3684	*MAT*a *ade2 his3 leu2 trp1 ura3 HMR tRNA +19 HMR*-*ADE2 BRF1-3XFLAG*:*KanMX*	Donze Lab
DDY3688	*MAT*a *ade2 his3 leu2 trp1 ura3 HMR tRNA +19 HMR*-*ADE2 BRF1-3XFLAG*:*KanMX rpd3*Δ::*URA3*	Donze Lab
DDY3828	*MAT*a *ade2 his3 leu2 trp1 ura3 bre2*Δ::*LEU2*	Donze Lab
DDY5605	*MAT* **α** *ade2 his3 leu2 lys2*Δ *trp1 ura3 HMR*-*ADE2* VII-L-*URA3*-*TEL* ppr1Δ::*TRP1 rpd3*Δ::KanMX Tn:*LEU2*:*ngg1*	This study
DDY5609	*MAT* **α** *ade2 his3 leu2 lys2*Δ *trp1 ura3 VII-L-URA3-TEL ppr1*Δ::*TRP1 HMR-ADE2 rpd3*Δ::*Kan hst3*Δ::*LEU2*	This study
DDY5657	*MATa ade2 his3 leu2 lys2*Δ *trp1 ura3 HMR-ADE2 rpd3*Δ::*Kan hst1*Δ::*LEU2*	This study
DDY5675	*MAT* **α** *ade2 his3 leu2 lys2*Δ *trp1 ura3 HMR-ADE2 rpd3*Δ::*Kan hst1*Δ::*LEU2 hos2*::*TRP1*	This study
DDY5625	*MAT* **α** *ade2 his3 leu2 lys2*Δ *trp1 ura3 HMR ADE2* VII-L-*URA3*-*TEL ppr1*Δ::*TRP1 rpd3*Δ::KanMX Tn:*LEU2*:*set1*	This study
DDY5626	*MAT* **α** *ade2 his3 leu2 lys2*Δ *trp1 ura3 HMR*-*ADE2* VII-L-*URA3*-*TEL ppr1*Δ::*TRP1 rpd3*Δ::KanMX Tn:*LEU2*:*bre2*	This study
DDY5632	*MAT* **α** *ade2 his3 leu2 lys2*Δ *trp1 ura3 HMR*-*ADE2* VII-L-*URA3*-TEL *ppr1*Δ::*TRP1 rpd3*Δ::KanMX Tn:*LEU2*:*set1*	This study
DDY5635	*MAT* **α** *ade2 his3 leu2 lys2*Δ *trp1 ura3 HMR*-*ADE2* VII-L-*URA3*-*TEL ppr1*Δ::*TRP1 rpd3*Δ::KanMX Tn:*LEU2*:*bre2*	This study
DDY5636	*MAT* **α** *ade2 his3 leu2 lys2*Δ *trp1 ura3 HMR*-*ADE2 rpd3*Δ::KanMX	This study
DDY5639	*MAT* **α** *ade2 his3 leu2 lys2*Δ *trp1 ura3 HMR ADE2* VII-L-*URA3*-*TEL ppr1*Δ::*TRP1 rpd3*Δ::KanMX	This study
DDY5640	*MAT* **α** *ade2 his3 leu2 lys2*Δ *trp1 ura3 HMR*-*ADE2* VII-L-*URA3*-*TEL ppr1*Δ::*TRP1 rpd3*Δ::KanMX Tn:*LEU2*:*sir4*	This study
DDY5641	*MAT* **α** *ade2 his3 leu2 lys2*Δ *trp1 ura3 HMR*-*ADE2* VII-L-*URA3*-*TEL ppr1*Δ::*TRP1 rpd3*Δ::KanMX Tn:*LEU2*:*sir4*	This study
DDY5642	*MAT* **α** *ade2 his3 leu2 trp1 ura3 HMR*-*ADE2 rpd3*Δ::KanMX Tn:*LEU2*:*bre1*	This study
DDY5664	*MAT* **α** *ade2 his3 leu2 trp1 ura3 HMR*-*ADE2 rpd3*Δ::KanMX Tn:*LEU2*:*bre1*	This study
DDY5665	*MAT* **α** *ade2 his3 leu2 trp1 ura3 HMR*-*ADE2 rpd3*Δ::KanMX	This study
DDY5676	*MAT* **α** *ade2 his3 leu2 lys2*Δ *trp1 ura3 HMR*-*ADE2* VII-L-*URA3*-*TEL ppr1*Δ::*TRP1 rpd3*Δ::KanMX *bre1*Δ::*LEU2*	This study
DDY5681	*MAT* **α** *his3 leu2 trp1 ura3 HMR*ΔI + tDNA Barrier at *a2* Tn:*LEU2*:*rpd3*	This study
DDY5683	*MAT* **α** *ade2 his3 leu2 lys2*Δ *trp1 ura3 HMR*ΔI + tDNA Barrier at *a2* Tn:*LEU2*:*rpd3 bre2*Δ::*LEU2*	This study
DDY5690	*MAT* **α** *ade2 his3 leu2 lys2*Δ *trp1 ura3 HMR*-*ADE2* VII-L-*URA3*-*TEL ppr1*Δ::*TRP1 rpd3*Δ::KanMX Tn:*LEU2*:*bre2*	This study
DDY5691	*MAT* **α** *ade2 his3 leu2 trp1 ura3 HMR*-*ADE2 rpd3*Δ::KanMX Tn:*LEU2*:set1	This study
DDY5692	*MAT* **α** *ade2 his3 leu2 trp1 ura3 HMR*-*ADE2 rpd3*Δ::KanMX Tn:*LEU2*:*bre2*	This study
DDY5697	*MAT* **α** *ade2 his3 leu2 trp1 ura3 HMR*-*ADE2 rpd3*Δ::KanMX *set1*Δ::*LEU2*	This study
DDY5698	*MAT* **α** *ade2 his3 leu2 trp1 ura3 HMR*-*ADE2 rpd3*Δ::KanMX *set1*Δ::*LEU2*	This study
DDY5699	*MAT* **α** *ade2 his3 leu2 trp1 ura3 HMR*-*ADE2 rpd3*Δ::KanMX *bre1*Δ::*LEU2*	This study
DDY5700	*MAT* **α** *ade2 his3 leu2 trp1 ura3 HMR*-*ADE2 rpd3*Δ::KanMX *bre2*Δ::*LEU2*	This study
DDY5701	*MAT* **α** *ade2 his3 leu2 trp1 ura3 HMR*-*ADE2 rpd3*Δ::KanMX *bre2*Δ::*LEU2*	This study

All strains are isogenic to *S. cerevisiae* W303-1A with the genotype ade2-1 his3-11,15 leu2-3,112 trp1-1 ura3-1 can1-100. Lysine auxotrophic W303 derivatives have a complete deletion of the *LYS2* ORF (*lys2*Δ).

**Table 3:** Oligonucleotides used in this study

Oligo #	Sequence	Description
DDO-045	CGCCAGGGTTTTCCCAGTCACGAC	M13 -47, anchor bubble PCR and sequencing
DDO-046	GAAGGAGAGGACGCTGTCTGTCGAAGGTAAGGAACGG	Anchor bubble adaptor reverse
	ACGAGAGAAGGGAGAG	
DDO-047	GACTCTCCCTTCTCGAATCGTAACCGTTCGTACGAGAAT	Anchor bubble adaptor forward
	CGCTGTCCTCTCCTT	
DDO-048	CGAATCGTAACCGTTCGTACGAGAATCGCT	Anchor bubble PCR
DDO-198	GCACTCTCAGTACAATCTGC	pRS universal RC, upstream
DDO-199	CCGCACAGATGCGTAAGGAG	pRS universal RC, downstream
DDO-2155	TCAGAAGGTGCTCTCGAGATATCAACTCAGAGCGTATAGG	RPD3 clone XHOI upstream
DDO-2156	TCGATGATTTAAGCGGCCGCAGTCATTTACCCAGGCGTG	RPD3 clone NOTI downstream
DDO-2167	CTAGCATAGGTAACATTCCTTATTTGTTGAATCTTTATAAGA	SET1 pRS delete top
	GGTCTCTGCGTTTAGAGAGCAGATTGTACTGAGAGTGC	
DDO-2168	TTTGCTGGAAAGCAACGATATGTTAAATCAGGAAGCTCCAA	SET1 pRS delete bottom
	ACAAATCAATGTATCATCGCTCCTTACGCATCTGTGCGG	
DDO-2169	ACGTAAACAACGGCAAAGAAC	SET1 upstream check 326 BP
DDO-2170	TCCGTGGCCTTTACGTTTTC	SET1 downstream check 312 BP
DDO-912	AGGGCTTTCACCGTTTTTATGCTAATCGTGCTAGCTGATAA	BRE1 pRS KO upstream
	TAATCAGATGCAGATTGTACTGAGAGTGC	
DDO-913	TATGTGGAGGATATAACACAAACAGTGGAAAAGTGGTAGAA	BRE1 pRS KO downstream
	TAATTAGTACTCCTTACGCATCTGTGCGG	
DDO-914	AATATTGGGAAAATCACTGGTG	BRE1 KO upstream 386 BP
DDO-915	GAACAAGCGCGATTAAGGTC	BRE1 KO downstream check 327 BP
DDO-925	GATAAAGGTGGCCATAATTGGACGAAGACAAATAATTCACT	BRE2 pRS KO upstream
	TCCTTAATAGCAGATTGTACTGAGAGTGC	
DDO-926	TAAGAAACACACTTTCAGTGTGTTTTAATTATTCTTCTTTGA	BRE2 pRS KO downstream
	ATGCTGCTCTCCTTACGCATCTGTGCGG	
DDO-2183	CACCTCTTACAGCTAGGAAAC	BRE2 upstream check 425 BP
DDO-928	AGGAGCTGTTATTTAGTCGGTCG	BRE2 KO downstream check 390 BP
DDO-681	GCAAACCAACTTTCTAGTATTC	HMR a2
DDO-951	GTCCATCGTCATCTGAAAAATAATG	HMR at tDNA
DDO-59	CATACTCGAAGGGTAGTTGG	Chromosome III tRNA Brf1 ChIP-A top
DDO-60	GATTTTTCCATTCGCCATGC	Chromosome III tRNA Brf1 ChIP-A bottom
DDO-482	GGCGATATAATTTATCATGTTTTGG	HMR I silencer Brf1 ChIP-B top
DDO-483	TCTCTAACTTCGTTGACAAATTTTC	HMR I silencer Brf1 ChIP-B bottom
DDO-484	CCAATTCCGCATCTGCAGATTACTT	HMR tDNA Brf1 ChIP-C top
DDO-485	TTCATTATTTTTCAGATGACGATGG	HMR tDNA Brf1 ChIP-C bottom
DDO-1027	CATAACACTGACATCTTTAACAAC	ADE2 promoter Brf1 ChIP-D top
DDO-1028	CTAATATACCAACTGTTCTAGAATC	ADE2 promoter Brf1 ChIP-D bottom
DDO-767	TCCGCAAGATTACTGCGGCTGCTTCC	+19 tRNA specific Northern probe

Parent *HMR*-*ADE2 rpd3* reporter strains DDY3133 and DDY2973 (Table 2) were constructed by crossing DDY814 (Jambunathan *et al.* 2005) with *rpd3*Δ::KanMX strains. When grown in media containing suboptimal levels of exogenous adenine, yeast colonies deficient in adenine biosynthesis accumulate a red pigment derived from the Ade2p substrate (Roman 1956). *HMR*-*ADE2 rpd3*Δ strains grow as red colonies on agar minimal media (Yeast nitrogen base, U.S. Biologicals Y2025) containing 45 µg/ml adenine (15% of the normal 300 µg/ml level used) due to increased spreading of silencing that represses the ectopic *ADE2* allele at *HMR*. This sufficient but suboptimal level of adenine is critical to the colony color assay, as lower levels (less than 15 µg/ml) of adenine activate the *ADE2* promoter to overcome silencing and higher levels (over 90 µg/ml) feedback inhibit the pathway, both leading to white colony growth. Colonies typically were grown for 3 days at 30°C, then plates were held at 4°C for 3–4 days to obtain optimum pigmentation color prior to photographing on a dissecting microscope. Slow-growing *rpd3 hst1 hos2* mutants were grown for 5 days at 25°C to obtain optimum pigmentation.

The yeast transposon mutagenized library was obtained from Mike Snyder (Stanford University), and mutagenesis was performed as described (Burns *et al.* 1994; Ross-Macdonald *et al.* 1997, 1999a, 1999b). Transformations were plated on minimal media lacking leucine and containing 15% adenine (minus leu-15% ade), and rare white Leu+ colonies were picked and restreaked on minus leu-15% ade plates to verify the stability of the phenotype. Identification of mutagenized genes was performed using the vectorette PCR method as described (Ross-Macdonald *et al.* 1999b). Primary isolates were verified as single transposon insertions by backcrossing with DDY814 to verify 2:2 segregation of the *LEU2* marked transposon insertion, and complete cosegregation of Leu+ Kan+ markers with white and Leu− Kan+ with red colony phenotypes.

Direct deletion of *SET1*, *BRE1*, and *BRE2* was performed by standard yeast genetics procedures by amplifying the *LEU2* gene from plasmid pRS405 (Sikorski and Hieter 1989) with primers containing homology to the immediate upstream and downstream regions of each gene (listed and described in Table 3). After multiple attempts at directly deleting these genes in the *rpd3*Δ background yielded only one deletion strain, PCR products were transformed into strain DDY2973 containing *URA3*-marked plasmid pDD1340 expressing wild-type *RPD3*. We speculated that our haploid *rpd3*Δ strains were mitotic recombination deficient, which has been demonstrated for homozygous *rpd3*Δ diploid yeast (Dora *et al.* 1999). We estimated an approximately 10-fold increase in recombination efficiency in *RPD3* plasmid-containing transformations. Leu+ transformants were selected on minus leu-15% ade plates, and white colonies were restreaked to 5-FOA (5-fluoroorotic acid) plates to isolate Leu+ colonies that lost the *URA3*-marked *RPD3* plasmid. These 5-FOA^R^*rpd3*Δ isolates were confirmed for proper *SET1*, *BRE1*, or *BRE2* gene deletion by PCR on each end (confirmation oligos listed in Table 3), then restreaked on minus leu-15% ade plates to reconfirm the white colony phenotype. Plasmid pDD1340 was constructed by PCR amplification of *RPD3* from ∼240 bp upstream of the ORF to ∼350 bp downstream with oligos DDO-2155 and DDO-2156 and Q5 polymerase (New England Biolabs), using an *RPD3*-containing genomic library plasmid (with a 12 kilobase insert) as template. The PCR product was digested with *Xho* I and *Not* I then cloned into *URA3* vector pRS416 (Sikorski and Hieter 1989) also digested with *Xho* I and *Not* I. Plasmid isolates were confirmed by transformation into DDY2973 and complementation of the red colony phenotype back to white.

Mating assays were performed as previously described (Donze and Kamakaka 2001; Jambunathan *et al.* 2005). To generate the strains for the mating assay in Figure 5, strain DDY1344 (Jambunathan *et al.* 2005) was mated to DDY3828, sporulated, and tetrads dissected. Since the *HMR*-barrier locus is not marked and both *bre2*Δ and Tn:*rpd3* alleles were marked with *LEU2*, genotypes were verified by both *LEU2* segregation patterns and PCR. *HMR*ΔI-barrier loci were confirmed by PCR with oligonucleotides DDO-681 and DDO-951, and ± *bre2*Δ::*LEU2* alleles verified by PCR with DDO-928+DDO-199. Double *rpd3 bre2* strains were identified by 2:2 segregation of leucine prototrophy and *bre2*Δ confirmed by PCR. Deletion of only *RPD3* was confirmed as Leu+ isolates that were negative for the *bre2* deletion.

Northern blot analysis of the 19 base pair marked *HMR*-tRNA was performed as described (Braglia 2007). Chromatin immunoprecipitation of FLAG-tagged *BRF1* was also performed as previously described (Rusché *et al.* 2002; Simms *et al.* 2008).

All yeast strains and plasmids described in this study are available on request. The authors affirm that all data necessary for confirming the conclusions of this article are represented fully within the article, its tables and figures and the cited literature.

### Screen design

**Figure 1:**
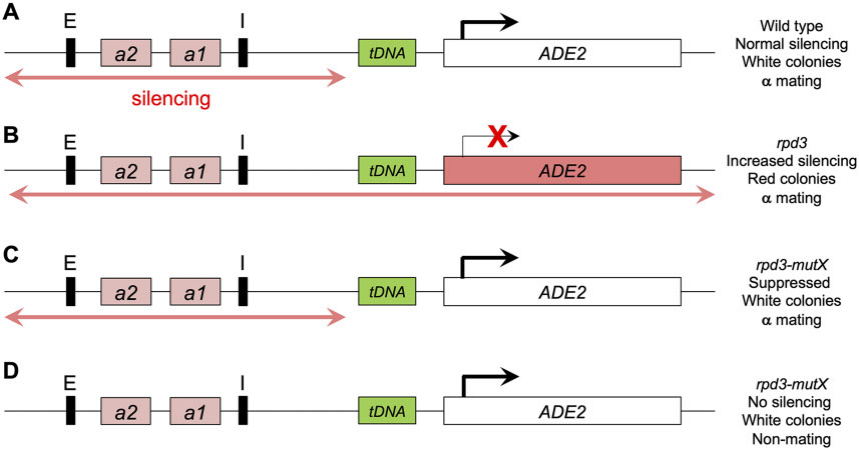
Schematic diagram of screen to identify suppressors of increased silencing in *rpd3* mutants. (A**)** *MAT***α** strains with a defective native *ade2-1* allele and containing functional *ADE2* integrated downstream of the *HMR-tDNA* barrier express *ADE2* and grow as white colonies. (B**)** Deletion of *RPD3* results in increased extended silencing through the barrier that represses *ADE2* expression and results in red colonies on minimal media containing suboptimal amounts of adenine. (C**)** *LEU2*-marked transposon mutagenesis of *rpd3*Δ strains identified rare Leu+ white colonies on media lacking leucine and containing suboptimal adenine. (D**)** White colonies were screened in a mating assay to identify and exclude unwanted nonmating isolates that have completely lost silencing (*e.g.*, those with transposon insertions in the *SIR* genes).

To identify suppressors that reverse *rpd3*Δ mediated increased silencing through the tDNA barrier at the *HMR* domain, we employed a colony color assay that indicates spreading of silencing beyond the tDNA at *HMR*. The strategy of the screen is schematically depicted in Figure 1. Previously engineered yeast strains with the nonfunctional *ade2-1* allele at the endogenous locus and containing a functional ectopic *ADE2* gene inserted downstream of the boundary results in normal *ADE2* expression and white colony growth (DDY814). We previously demonstrated that *rpd3* mutants result in spread of silencing beyond the boundary, resulting in repression of *ADE2* and growth as red colonies on 15% ade media (Jambunathan *et al.* 2005; Simms *et al.* 2008). Strains DDY3133 and DDY2973 (Table 2) were mutagenized using *LEU2*-marked transposon mutagenesis and plated on minus leu-15% ade glucose minimal agar. White Leu+ colonies were identified as containing transposon insertions in genes that are potential suppressors of extended *rpd3*Δ silencing.

Leu+ transposon insertion isolates were verified by several tests before identification of the transposon insertions, which led to only a fraction of the primary isolates being pursued. Initially, each white colony was restreaked on minus leu-15% ade plates to verify the maintenance of phenotype, and multiple isolates initially appearing as white reverted back to red growth and were not pursued. Isolates that displayed a consistent white to very light pink colony phenotype were tested for mating against a *MATa* tester strain (DDY20), as complete loss of silencing (*e.g.*, transposon insertions into one of the *SIR* genes) results in a nonmating phenotype. Out of 71 primary white colony isolates, 18 were nonmating. Two randomly selected nonmating isolates were found to have independent transposon insertions within the *SIR4* gene (DDY5640 and DDY5641). Transposon insertion sites were determined by the anchor bubble ligation-vectorette PCR method (Ross-Macdonald *et al.* 1999b), and insertion coordinates in Table 1 correspond to the current update of the *Saccharomyces* Genome Database (Cherry *et al.* 2012). Multiple isolates did not yield a PCR product and the insertion site could not be identified.

## Results

### Transposon mutagenesis screen for suppressors of *rpd3*Δ extended silencing yields hits predominately in the histone H3K4 methylation pathway

**Figure 2:**
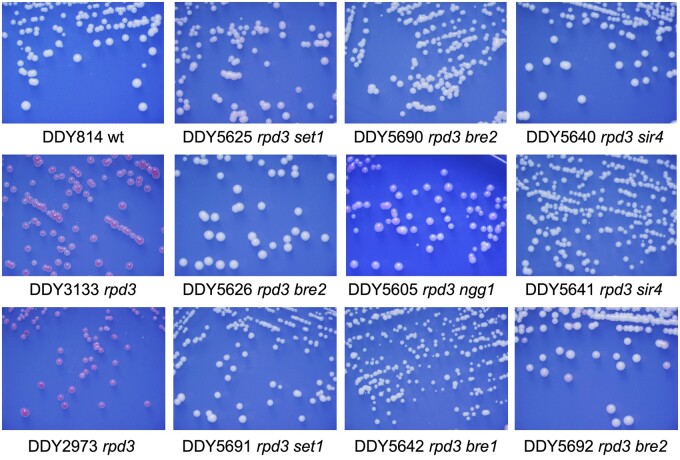
Representative primary Leu+ isolates from mutagenesis of *HMR-ADE2 rpd3*Δ strains were restreaked multiple times for single colonies to verify stable propagation of the white colony phenotype. A single isolate containing the insertion in *NGG1* gave rise to a variegated light pink phenotype. Additionally, isolates DDY5625 (*set1*) and DDY5692 (*bre2*) gave rise to rare very light pink colonies upon multiple restreaks of white colonies. DDY5640 and DDY5641 are two randomly selected Leu+ nonmating white colony isolates, and both contained different transposon insertions in the *SIR4* gene consistent with a complete loss of silencing.

Table 1 lists the identified Leu+ transposon mutagenized strains that lost the *rpd3*Δ extended silencing phenotype and grew as white colonies and retained a normal mating phenotype (therefore, did not lose normal *SIR*-mediated silencing), plus two randomly selected nonmating isolates. Interestingly, most of the identified mutants were in the histone H3K4 methylation pathway, including multiple hits in each *BRE1*, *BRE2*, and *SET1*. An additional insertion in *NGG1*, which codes for a component of the SAGA coactivator complex resulted in a very light pink and variegated phenotype, suggesting a partial suppression of increased *rpd3*Δ mediated silencing. However, this insertion also resulted in a slow growth phenotype, and it is not clear whether slow growth affects the accumulation of the pigment in this reporter assay. Figure 2 shows restreaked colonies grown on minus leu-15% ade plates derived from selected primary isolated mutants.

As described above, two randomly selected nonmating isolates were found to have transposon insertions within the *SIR4* gene. Identification of white colony isolates containing insertions within the *SIR* genes was expected, as they completely abolish *HMR* silencing. Additional expected insertions were identified in adenine biosynthesis pathway genes, including *ADE3*, *ADE4*, *ADE5,7*, and *ADE6*, as second adenine pathway mutations are known to suppress the *ade2* red colony phenotype (Roman 1956).

To verify single transposon insertions and cosegregation of the insertion with the white colony phenotype, selected primary isolates were backcrossed to strain DDY814 which contains the *HMR*-*ADE2* marker gene. For each of the *NGG1*, *SET1*, *BRE1*, and *BRE2* insertions, tetrad analysis verified that the *LEU2* marked transposon segregated 2:2 verifying single insertions (unpublished data).

**Figure 3:**
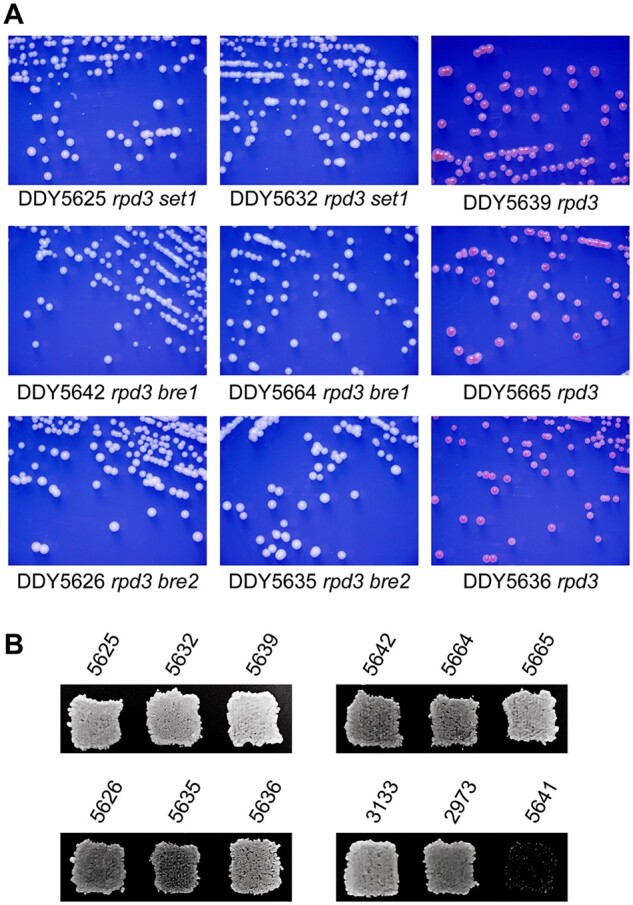
Colony color and mating phenotype of primary transposon mutants and haploid segregants after backcrossing to strain DDY814. (A**)** For each cross, the suppression phenotype bred true, as all double mutant segregants (Leu+ Kan+) produced white colonies, and segregants containing only the *rpd3*Δ::KanMX allele produced red colonies. Selected isolates were streaked on media lacking leucine and containing suboptimal adenine as in Figure 2. (B**)** The same *his3* isolates were patched to YPD plates and after overnight growth were replica plated to a lawn of strain DDY20 (*his4*) on yeast minimal media lacking histidine. Each strain mated, confirming normal *HMR* silencing is not lost due to the second mutations. Parent strains DDY3133 and DDY2973 were also confirmed as mating, and DDY5641 containing a transposon insertion in *SIR4* was included as a nonmating control.

Individual haploid strains derived from these backcrosses were consistent with the suppression phenotype observed in the primary isolates: all haploid segregants containing only the *rpd3*Δ::KanMX allele grew as red colonies on 15% ade media, and those with both *rpd3*Δ::KanMX and Tn:*LEU2* insertions in *SET1*, *BRE1*, or *BRE2* grew as white colonies (Figure 3A) and retained their mating phenotype (examples shown in Figure 3B). These results confirm that loss of the H3K4 methylation pathway suppresses the increased silencing phenotype of *rpd3* mutants without completely abolishing silencing at the *HMR* locus. One strain containing a transposon insertion into *SIR4* was included in the assay as a nonmating control (DDY5641, Figure 3B).

**Figure 4:**
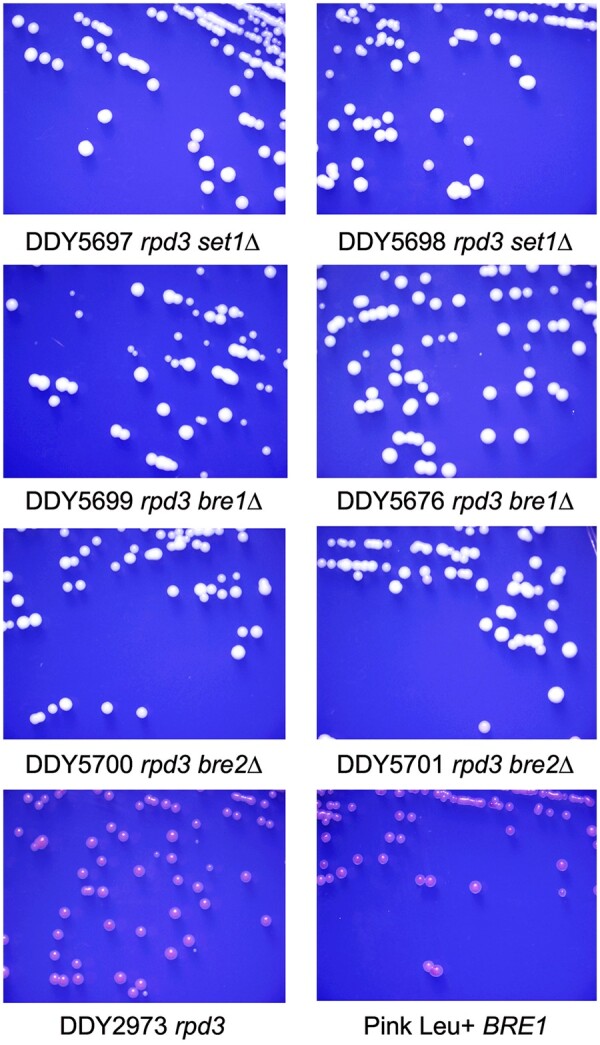
Confirmation of suppression phenotypes by direct knockout of candidate genes. Parent strain DDY2973 was transformed with an *LEU2* fragment containing homology to the flanking regions of each designated gene and plated on media lacking leucine and containing suboptimal adenine. White colonies were confirmed for the deletions by PCR at both ends of the deleted gene-*LEU2* junctions. Confirmed isolates were restreaked as in Figures 2 and 3, and one red colony from the primary *BRE1* transformation plate that tested negative for the deletion was included as an additional red control.

As an additional verification of suppression, we directly deleted *SET1*, *BRE1*, or *BRE2* in the parent strain DDY2973. White colonies from these knockout transformations were verified for the gene deletions by PCR on both ends of the *LEU2* marker gene and were streaked on minus leu-15% ade plates. Figure 4 shows two independent isolates of each knockout, and each grew as white colonies on the indicator media. As a control, a randomly selected Leu+ isolate from *bre1*Δ transformations that was not integrated at *BRE1* (likely recombined at the *leu2-3,112* locus) maintained the red colony color of the parent strain.

**Figure 5:**
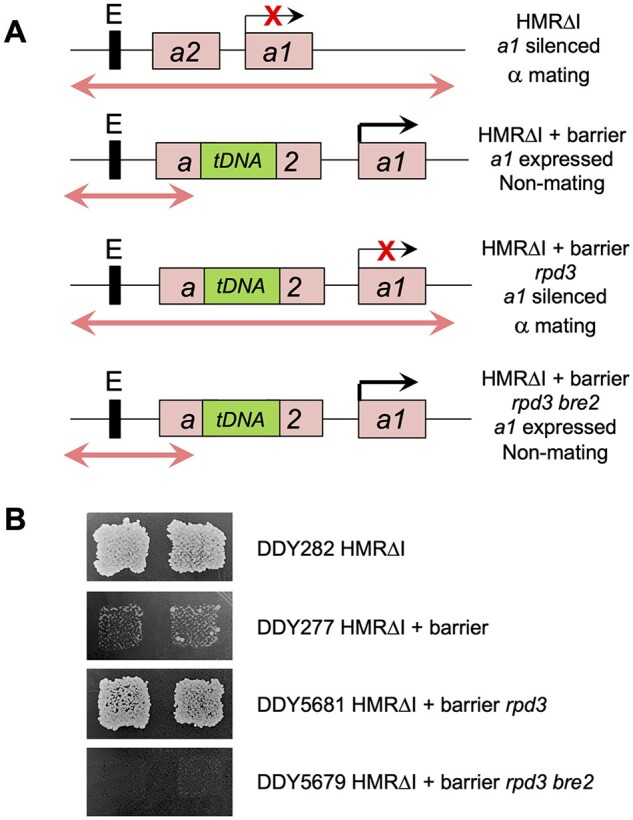
Alternate reporter assay for suppression of extended silencing phenotype. (A**)** Known and predicted phenotypes for each reporter strain are depicted as in Figure 1. Silencing of *a1* in the *MAT***α** background allows normal mating and growth on minimal media lacking histidine, while expression of *a1* results in a nonmating phenotype. (B**)** *MAT***α** *his3* reporter strains were patched onto YPD plates and grown overnight, then replica plated onto a lawn of strain DDY20 (*MAT*a *his4*) on YMD plates lacking histidine. Only mated diploid cells complemented for histidine biosynthesis grow. DDY282 lacks the barrier tDNA sequence, so *a1* is silenced in this positive control for mating. Insertion of the barrier in DDY277 blocks propagation of silencing, allowing expression of *a1* in the *MAT***α** cells to impart a nonmating phenotype. DDY5681 is identical to DDY277 except for the deletion of *RPD3*, resulting in the spread of silencing through the barrier to silence *a1* and allow mating. The transposon insertion in *BRE2* in DDY5679 suppresses the extended silencing caused by the deletion of *RPD3*, resulting in the expression of *a1* and the nonmating phenotype.

As a final verification that mutations in the SET1/COMPASS pathway specifically suppress the increased *rpd3*Δ mediated silencing at *HMR* and was not simply affecting the *ADE2* promoter of the reporter gene or the expression of other *ADE* pathway genes, we used a silencing-dependent mating assay that relies on the repression of a different promoter (schematically depicted in Figure 5A). Our original increased silencing *rpd3* mutants were identified using this mating assay (Jambunathan *et al.* 2005) where the *HMR*-tDNA barrier is inserted between the *HMR*-E silencer and the *HMR*-*a1* gene in a *MAT***α** background. Deletion of the *HMR*-I silencer and the barrier tDNA still allows repression of *a1* by the *HMR*-E silencer alone in the parent *MAT***α** strain, as the cells exhibited a normal mating phenotype (Figure 5B, DDY282). In strain DDY277, blocking of silencing by the ectopically inserted tDNA barrier resulted in the expression of the *a1* gene in the *MAT***α** background resulting in a nonmating phenotype. The increased spread of silencing in *rpd3*Δ mutants again repressed *a1* and allowed mating (DDY5681), and this phenotype was reversed in a strain containing mutations in both *rpd3 and bre2* which became nonmating (DDY5679).

**Figure 6:**
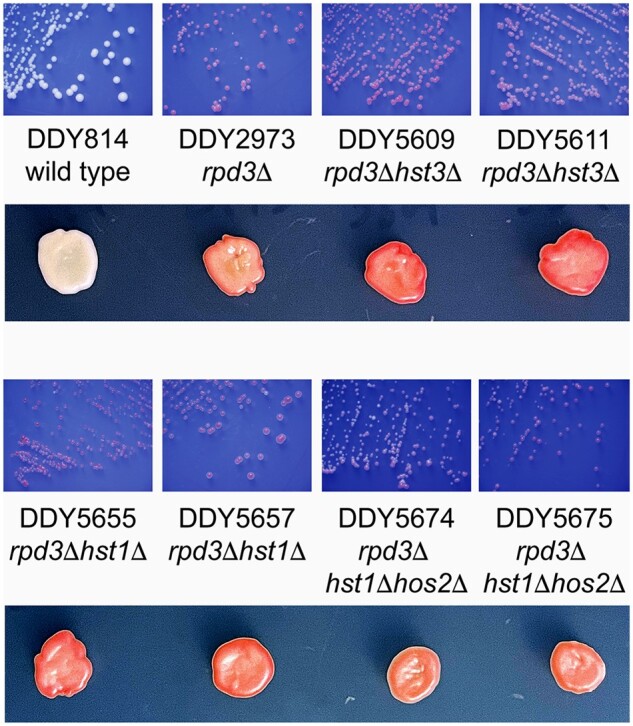
Deletion of known effectors of H3K4me mediated gene repression do not affect extended *rpd3*Δ silencing. Strains were constructed in the *rpd3*Δ background to have deletions of HDAC genes *HST3*, *HST1*, or both *HST1 and HOS2*. Since *hst1*Δ*hos2*Δ strains grew slowly, patches of ∼5 mm diameter of each strain were made to obtain comparable growth to verify the red phenotype (lower panels).

The *SET1* pathway has been demonstrated to repress target genes by promoting the recruitment of alternative HDACs, namely Hst3p, or a combination of Hst1p and Hos2p (Kim and Buratowski 2009; Jaiswal *et al.* 2017). We tested the potential role of these HDACs as possible mediators of extended silencing in *rpd3* mutants by constructing deletions in these genes in our *rpd3*Δ reporter strains. Deletion of *HST3*, *HST1*, or both *HST1 and HOS2* still yielded pigmented colonies as shown in Figure 6. Strains deleted for *HST1 and HOS2* are slow growing, which partially reduces the accumulation of pigment within single colonies, but the ectopic *ADE2* marker gene is still repressed in these strains as seen in the patched growth (Figure 6, lower panels) indicating that increased silencing is not mediated by these HDACs.

**Figure 7:**
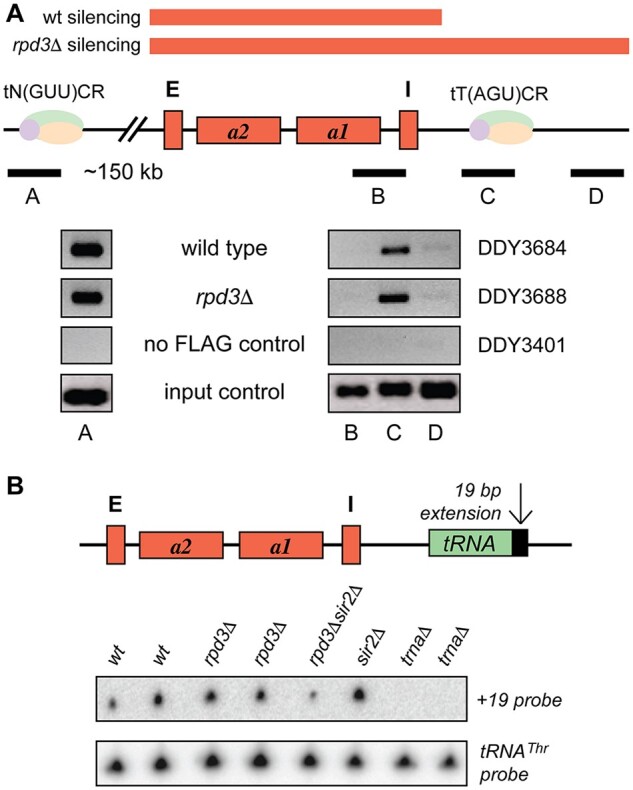
Binding of RNA Polymerase III transcription complex and *HMR*-tRNA expression are not affected by increased *rpd3*Δ silencing. (A) Chromatin immunoprecipitation was performed using anti-FLAG antibody in wild type and *rpd3*Δ strains containing FLAG-tagged Brf1p. PCR primer sets distal to (B and D) and overlapping the *HMR*-tDNA (C) were used to determine Pol III complex formation. Primer set A surrounds a tDNA distal to the *HMR* domain and was used as a positive control. A strain lacking the FLAG epitope on Brf1p was used as a negative control. (B**)** Northern blot analysis of a marked *HMR*-tDNA confirms its expression in *rpd3*Δ mutants. The *HMR*-tRNA transcript is detected using a complementary oligonucleotide probe specific to the 19 base pair extension (DDO-767, Table 3) to distinguish it from transcripts emanating the other seven copies of this tRNA^Thr^ isoacceptor. Strains deleted for the *HMR*-tDNA show no signal, confirming the specificity of the assay. The blot was stripped and reprobed with a bulk tRNA^Thr^ 76 base oligonucleotide probe (complementary to the tRNA^Thr^(AGU)C, SGD YNCC0014W final processed transcript) as a loading control. Strains used are (left to right) DDY3401, 3402, 3403, 3404, 3396, 3398, 465, and 466.

Finally, we addressed the question of whether inactivation of *RPD3* function affects the tDNA barrier itself. We performed chromatin immunoprecipitation to assay RNA Polymerase III complex formation at the *HMR*-tDNA using strains expressing FLAG-tagged Brf1p, which is part of the Pol III complex. Results in Figure 7A show no significant difference in Pol III complex formation in the *rpd3*Δ background, despite silencing spreading through or around the tDNA (see *Discussion*). We also performed Northern blot analysis to assay relative transcription levels of the *HMR*-tDNA using strains specifically marked with a 19 base pair extension to distinguish *HMR*-tRNA transcripts from those encoded by the seven other copies of this tRNA^Thr^ isoacceptor in *S. cerevisiae* (Donze and Kamakaka 2001; Braglia *et al.* 2007). The results in Figure 7B showed no difference in *HMR*-tRNA or bulk tRNA^Thr^ expression in *rpd3*Δ strains versus wild type. These results suggest that loss of *RPD3* function does not grossly affect Pol III complex formation or tRNA transcription at *HMR* or other tRNA^Thr^ loci.

## Discussion

Enhancement of Sir-protein mediated silencing by loss of *RPD3* function is an intriguing paradox, made more interesting by the numerous studies that uncovered this phenomenon affecting all three Sir-protein repressed domains in *S. cerevisiae* as described in the introduction. To identify genes that might be misregulated in *rpd3* mutants or otherwise involved in the extended silencing phenotype, we conducted a suppressor screen using transposon mutagenesis. Interestingly, most of the hits were in the histone H3K4 methylation pathway in *SET1*, *BRE1*, and *BRE2*. It is of note that we initially began this screen using a more cumbersome ultraviolet light induced mutagenesis procedure to isolate white colonies, followed by yeast plasmid library complementation of individual strains back to the red phenotype. Before switching to the transposon mutagenesis procedure, we isolated complementing plasmids containing *BRE1 and BRE2* (R. A. Kleinschmidt, PhD dissertation, http://digitalcommons.lsu.edu.libezp.lib.lsu.edu/do/search/?q=etd-07052011-094524/), consistent with our findings reported here.

COMPASS (Complex Proteins Associated with Set1) is a conserved multiprotein complex recruited to chromatin to methylate H3K4 (and possibly other substrates) and contains both Set1p and Bre2p (Miller *et al.* 2001; Cenik and Shilatifard 2021). *SET1* encodes the sole H3K4 methyltransferase activity in *S. cerevisiae* which is responsible for mono-, di-, and trimethylation of this residue. *BRE2* encodes a key structural factor within this complex that is also required for methylation (South *et al.* 2010; Hsu *et al.* 2018; Qu *et al.* 2018). Recruitment of COMPASS and subsequent H3K4 methylation requires prior histone H2B ubiquitylation mediated by the Bre1p E3-ubiquitin ligase complexed with the Rad6p ubiquitin-conjugating enzyme (Dover *et al.* 2002; Sun and Allis 2002). While initially thought to promote transcriptionally active chromatin structures as is acetylation due to its prevalence near active promoters, H3K4 methylation was also found to be associated with repression of a subset of yeast genes (Briggs *et al.* 2001; Bryk *et al.* 2002; Kim and Buratowski 2009). Interestingly, although the mutagenesis procedure was calculated to be saturating, we did not find transposon insertions in *RAD6*, or in genes involved in Dot1p methylation of H3K79 which also requires H2B ubiquitylation (Briggs *et al.* 2002).

We also looked at several possible mechanisms for the formation and reversal of *rpd3*Δ mediated extended silencing at *HMR* by mutations that affect the *SET1* pathway. Thurtle-Schmidt *et al.* (2016) also identified *rpd3* mutations as suppressors of defective silencing in a catalytically inactive *sir2*N345A mutant. In that study, restored silencing was mediated by the *SIR2* related Sirtuin HDAC Hst3p, as *rpd3 hst3* mutants did not suppress the *sir2* mutation. We deleted *HST3* in our *HMR*-*ADE2 rpd3*Δ strains, but the resulting strains maintained the red colony phenotype (Figure 6), ruling out a role for H3K4me targeting of Hst3p in our system. One difference here (and possibly in other studies) might be that in the Thurtle-Schmidt study, the *URA3* reporter gene used is located between the *HMR*-E and *HMR*-I silencers, while our *ADE2* reporter is downstream from the *HMR*-I silencer. This might reflect an interesting potential difference in the mechanisms of normal versus *rpd3*Δ-mediated silencing propagation between the silencers versus downstream of *HMR*-I in *rpd3* mutants. While several studies have described that mutation of the SET pathway weakens *SIR*-mediated silencing in yeast, most used reporter genes at telomeres and the rDNA locus or between the *HMR* or *HML* silencers. The maintenance of mating in our primary SET pathway mutants and their progeny from crosses (Figure 3) suggests that normal *HMR* silencing of the *a1* gene is not abolished in SET pathway mutants in the *rpd3*Δ background.

Another role of the COMPASS complex is in the repression of middle sporulation genes which is mediated by another Sirtuin HDAC Hst1p in complex with Sum1p and Rfm1p (Jaiswal *et al.* 2017). However, deletion of *HST1* in our *HMR*-*ADE2 rpd3*Δ strains again maintained the red colony phenotype (Figure 6). Another proposed mechanism of Set1p directed repression is through H3K4me recruitment of the *SET3* complex (Kim and Buratowski 2009), which contains two HDACs, encoded by *HST1 and HOS2*. Double deletion of these genes in our reporter strains again resulted in pigmented colonies (Figure 6). One other possible mechanism for suppression of extended silencing involves a study by Santos-Rosa *et al.* (2004) that reports a reduction of Sir-protein binding at heterochromatic loci in *set1* mutants. However, consistent with our suppressed strains maintaining mating, a closer examination of their data reveals that Sir3p association is reduced at *HML* and telomeres but not at *HMR* in *set1*C1068A and H3K4R mutants. Therefore, we hypothesize that another, yet uncharacterized mechanism of *SET1*-mediated repression might be at play when silencing extends beyond the tDNA barrier at *HMR* in *rpd3* mutants. In addition to effects on COMPASS complex recruitment and function at the *HMR* barrier region, interesting possibilities to be pursued are whether *rpd3* mutation affects nucleosome positioning, known to play a role in regulating silencing (Saxton and Rine 2020) or affects *HMR* origin firing known to be influenced by *SET1* through H3K37 methylation (Santos-Rosa *et al.* 2021).

Also, intriguing are our findings that while silencing spreads beyond the *HMR*-tDNA barrier in *rpd3* mutants, the Pol III complex remains bound to the tDNA sequence and remains active (Figure 7). This contrasts with a previous study where we demonstrated that loss of function of the nonhistone proteins Nhp6a and Nhp6b leads to spreading of silencing past the tDNA barrier and loss of transcription of the *HMR*-tDNA (Braglia *et al.* 2007). We hypothesize that in the absence of *RPD3* function, the tDNA is possibly somehow looped out to allow discontinuous spreading of heterochromatin around the actively transcribed tDNA. It will be interesting to test this hypothesis by chromatin conformation capture methods and to determine how loss of *RPD3* HDAC function allows formation of such a chromatin structure to allow silencing to bypass the barrier and how this spreading is then suppressed by loss of H3K4 methylation. Since the Pol III complex remains intact, this would also require a detailed analysis of potential differences in H3K4 methylation marks on nucleosomes immediately adjacent to the *HMR*-tDNA in wild type versus *rpd3* mutants.

So how does loss of *RPD3* function lead to increased silencing at *HMR*? One study suggests that *RPD3* acts as a barrier protein by removing the acetylated lysine substrate for Sir2p and inhibiting the local formation of the OAADPR (o-acetyl ADP-ribose) product of Sirtuin deacetylases which has been implicated in promoting silencing (Ehrentraut *et al.* 2010). However, an earlier study casts some doubt on this potential mechanism by demonstrating that *SIR*-mediated silencing can propagate via deacetylation by a nonsirtuin HDAC in the absence of local OAADPR production (Chou *et al.* 2008). However, consistent with a direct role of Rpd3p in barrier function we found in our bioinformatic analysis of published Rpd3p ChIP-seq data (McKnight and Tsukiyama 2015) that weak but consistent peaks of Rpd3p are present at numerous tDNAs in wild-type yeast, including at *HMR*. We are currently constructing yeast strains to confirm this potential association by conventional ChIP. Since *SIR*-mediated silencing in *S. cerevisiae* is modeled to propagate by sequential Sir2p deacetylation followed by Sir3p and Sir4p binding (Rusché *et al.* 2002), if Rpd3p is indeed localized at tDNAs it could contribute to barrier function by simply removing the acetyllysine docking sites for Sir2p activity over a range of nucleosomes near the *HMR*-tDNA. The subsequent loss of *RPD3* function would then allow Sir2p to promote the propagation of silencing on both sides of the tDNA by attaching to and deacetylating the nucleosomes normally targeted by Rpd3p. Why subsequent spreading of silencing in *rpd3* mutants is then dependent on the *SET1* pathway remains to be determined.

These studies are significant to gain a deeper understanding of the interplay and crosstalk of chromatin writers, erasers, and readers (Allis and Jenuwein 2016) but may also have relevance to current proposed applications of HDAC and other chromatin modification enzyme inhibitors in health care (West and Johnstone 2014; Ansari 2019; Bhat *et al.* 2021; Dang and Wei 2021). While therapeutic results have been seen using drugs that inhibit chromatin enzymes, the global effects of removing one chromatin-modifying activity on the myriad of others and all possible interactions and potential side-effects are not fully characterized. Other possibilities to consider are that chromatin-modifying enzymes have been shown to act on nonhistone targets (Toro and Watt 2020; Di Blasi *et al.* 2021), and some chromatin-modifying complexes have additional nonenzymatic functions (Morgan and Shilatifard 2020) which could also be relevant in our system. Therefore, an understanding of the downstream effects of changes in the activity of protein complexes containing chromatin writers, erasers, readers, and the individual modified target residues (on both nucleosomes and nonhistone proteins) continues to warrant further study at both the global level and at individual genomic loci.

## Data availability

All yeast strains and plasmids described in this study are available on request. The authors affirm that all data necessary for confirming the conclusions of this article are represented fully within the article, its tables and figures and the cited literature.
